# Optimization of Exoskeleton Assistance Function Based on Physics-Guided Dynamic Fusion Model

**DOI:** 10.3390/bioengineering13050531

**Published:** 2026-05-01

**Authors:** Haochen Tian, Jiaxin Wang, Shijie Guo, Feng Cao, Lei Liu

**Affiliations:** 1 Academy for Engineering & Technology, Fudan University, Shanghai 200433, China; 19110860025@fudan.edu.cn; 2 School of Mechanical Engineering, Hebei University of Technology, Tianjin 300401, China; wangjx@hebut.edu.cn (J.W.); 202332804007@stu.hebut.edu.cn (F.C.); 202131205073@stu.hebut.edu.cn (L.L.)

**Keywords:** soft exoskeleton, joint moment estimation, neural network, profile optimization, personalized assistance

## Abstract

Wearable lower-limb exoskeletons can enhance mobility, reduce metabolic cost, and aid rehabilitation. Effective human-exo cooperation requires personalized assistance profiles that match biomechanical principles. Existing methods often rely on fixed curves, involve complex tuning, and lack biomechanical interpretability. To address this, we propose a “Physics-guided perception and physiology-driven optimization” approach. First, a Physics-guided Dynamic Fusion Model (PDFM) is proposed, which integrates Newton–Euler dynamics, LSTM, and NTM to estimate multi-plane hip joint moments without ground reaction forces, employing biomechanical models as complementary fusion factors rather than the embedded hard constraints used in conventional physics-informed neural networks (PINNs). The model achieved correlation coefficients of 0.938, 0.924, and 0.929, and relative root mean square error (rRMSE) values of 5.29%, 9.79%, and 5.61%, in the sagittal, coronal, and transverse planes, respectively. These results outperformed all single-network baselines across all three anatomical planes. Second, an assistance profile derived from estimated moments is individually optimized using Bayesian optimization based on multi-muscle sEMG. Compared to no-exo walking, the optimized system reduced target muscle loading by 49.31% and metabolic cost by 14.75%; relative to the pre-optimized profile, the reductions were 23.64% and 5.74%, respectively. This work provides a laboratory-validated framework for personalized hip exoskeleton assistance in healthy adults, establishing a foundation for future clinical translation.

## 1. Introduction

Wearable lower-limb exoskeleton technology demonstrates significant potential in enhancing human locomotor performance, reducing metabolic cost during walking, and promoting neurological rehabilitation [1,2,3]. Among various joint assistance strategies, hip assistance has been proven to be more energy-efficient than others while also improving center-of-mass stability to some extent [4,5]. Realizing this advantage requires efficient and natural human–robot synergy, meaning the exoskeleton must precisely supplement or substitute the function of hip flexor/extensor muscles. Consequently, generating a personalized assistance profile that conforms to the intrinsic biomechanical laws of the human body is crucial. Many recent studies have employed pre-defined assistance profiles as reference templates for exoskeleton force/moment output [6,7]. This approach not only diminishes the biomechanical and physiological interpretability of the exoskeleton’s assistance effect but also complicates personalized adaptation from the initial stage of assistance planning [8]. To address these limitations, assistance strategies referencing the biological hip joint moment have emerged [9,10]. By matching the exoskeleton’s assistance moment to the user’s biological joint moment, these strategies effectively reduce the metabolic cost of walking through a decrease in muscular mechanical work [11].

Accurate acquisition of personalized hip joint moments is a prerequisite for implementing this assistance strategy. The gold standard for calculating hip joint moments is the Newton–Euler (N–E) inverse dynamics algorithm. However, its heavy reliance on ground reaction forces (GRF) severely limits its practicality in wearable applications [12]. In recent years, data-driven estimation methods, particularly those utilizing neural networks to directly learn the moment-mapping relationship from electromyography (EMG) signals and kinematic data, have reduced modeling and measurement complexity, offering a promising solution to eliminate the dependency on force plates [13]. While EMG signals provide physiological insights, strict sensor-wearing requirements (accurate positioning, resistance to displacement, and clean skin contact) significantly reduce their practicality during prolonged or vigorous activities. Consequently, kinematic data are less restrictive to measure, involve smaller data volumes, offer higher precision, and are simpler to operate. Furthermore, they can be acquired using wearable sensors, such as inertial measurement units (IMUs), integrated into the exoskeleton’s perception module, granting them higher usability in research on real-time joint moment estimation. In terms of model architecture, researchers have proposed hybrid models that leverage the complementary strengths of multiple networks to circumvent the accuracy and generalization constraints inherent to single neural network architectures [14]. Furthermore, researchers have found that different neural network architectures possess unique characteristics, making them suitable for different locomotion scenarios (e.g., walking, jumping, ascending/descending slopes) [15,16,17].

To overcome the aforementioned limitations—including the GRF dependency of the gold-standard inverse dynamics algorithm, the poor long-term practicality of EMG-driven approaches, the lack of physical interpretability inherent to data-driven neural network predictions, and the inherent bottlenecks in accuracy and generalizability of single neural network architectures—a Physics-guided Dynamic Fusion Model (PDFM) is first proposed in this paper. This model strategically integrates Newton–Euler dynamics, long short-term memory (LSTM) networks, and neural Turing machines (NTMs) to achieve multi-plane hip joint moment estimation without requiring ground reaction force measurements. Unlike conventional physics-informed neural networks (PINNs) that embed physical laws as hard constraints within the network architecture, the proposed method employs biomechanical models as complementary fusion factors to guide the data-driven estimation process.

After obtaining reliable moment estimates, assistance profiles for the exoskeleton can be generated through human-exo system modeling. However, precise adaptation to different users requires further personalized parameter optimization (e.g., assistance phase, amplitude, and duration) [18,19]. Among recent studies, methods based on metabolic rate optimization are frequently employed [20]. Yet, the extended duration of measurement and optimization in such protocols may induce user discomfort, potentially compromising the optimization outcome. In contrast, surface electromyography (sEMG)-based optimization, characterized by a shorter process and higher user comfort, has demonstrated promising feasibility for tuning joint assistance profiles [21]. Accordingly, we employ a Bayesian optimization framework driven by integrated electromyography (iEMG) across three target muscles (the Rectus Femoris, RF; Vastus Medialis Obliquus, VMO; Vastus Lateralis Obliquus, VLO) to achieve personalized calibration of the assistance amplitude. This approach circumvents the core drawbacks of conventional metabolic cost optimization strategies, namely their protracted experimental workflows and the associated discomfort for end users.

This study presents a moment-based assistance strategy for a soft hip exosuit optimized via multi-muscle iEMG and Bayesian optimization. The primary contributions are as follows: (1) a Physics-guided Dynamic Fusion Framework (PDFM) enabling high-accuracy multi-plane hip moment estimation without ground reaction forces; (2) a biomechanical conversion method transforming estimated sagittal moments into personalized assistance profiles through gait and human-exoskeleton dynamic modeling; (3) an efficient personalization protocol leveraging multi-muscle iEMG and Bayesian optimization for rapid parameter tuning; and (4) comprehensive pilot experiments in healthy adults validating the efficacy and feasibility of the proposed framework through convergent neuromuscular and metabolic metrics, as a prerequisite for future clinical translation. Results demonstrate that the PDFM achieves high-fidelity sagittal-plane estimation (correlation coefficient = 0.938, rRMSE = 5.29%), with the optimized system significantly reducing average iEMG by 23.64% and metabolic cost by 5.74% relative to pre-optimized conditions.

The rest of this article is organized as follows. Section 2 describes the experimental protocol, the proposed PDFM for hip moment estimation without ground reaction forces, and the assistance profile planning and optimization framework. Section 3 presents the model validation and wearable experimental results. Section 4 discusses the findings, limitations, and future work. The conclusions are given in Section 5.

## 2. Materials and Methods

The methodological framework is organized into two sequential stages aimed at establishing a biomechanically interpretable, ground-reaction-force-free pipeline for personalized hip assistance. First, we develop a Physics-guided Dynamic Fusion Model (PDFM) that estimates multi-plane hip joint moments using only kinematic data, thereby eliminating the dependency on ground reaction forces while preserving physiological consistency (Section 2.2). Second, we convert these subject-specific moment estimates into personalized exoskeleton-assistance profiles through human–exoskeleton dynamic modeling and Bayesian optimization (Section 2.3). The exoskeleton platform and experimental protocol are detailed in Section 2.1, and statistical methods are described in Section 2.4.

### 2.1. Exoskeleton Platform and Experimental Protocol

#### 2.1.1. The Soft Lower-Limb Exoskeleton Platform

**Figure 1:**
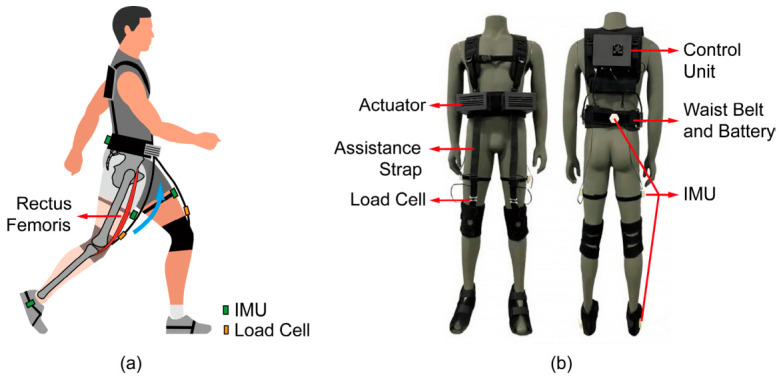
Soft hip flexion assist exoskeleton (based on the platform described in [22]). (**a**) Schematic diagram of the assistance principle, illustrating the actuator-driven tensile force transmitted via the assistance strap to the knee brace, producing a sagittal-plane hip flexion moment (blue arc) that mimics the rectus femoris (RF). The locations of the IMU and load cell are indicated. (**b**) Front and back views of the donned exoskeleton, showing the waist-mounted actuator, assistance strap, knee brace with integrated load cell, dorsal control unit, and battery-integrated waist belt.

The previously developed belt-type soft hip flexion exoskeleton was employed in this study (Figure 1). The waist-mounted actuator generates a controlled tensile force that is transmitted through flexible assistance straps to bilateral knee braces. This force vector produces a sagittal-plane hip flexion moment, thereby mimicking the mechanical function of the rectus femoris during the leg-lifting phase, as illustrated in Figure 1a. The total system mass is 2.7 kg, integrating sensing, control, actuation, and flexible transmission modules; the donning effect is shown in Figure 1b. IMUs distributed at the waist and lower-limb key positions are employed for gait-phase detection. A load cell integrated at the interface between the assistance strap and the knee brace provides force feedback for closed-loop control. The control unit and battery are secured on the dorsal trunk to minimize distal limb inertia. Compared with rigid-link exoskeletons, the flexible transmission structure accommodates soft-tissue deformation, effectively improving wearability and human–robot kinematic compatibility. A simplified assistance profile with an empirically set 35 N peak force was previously proposed, expressed as

(1) $$f_{h}=A_{h}\sin\left[\frac{\pi }{T_{s}}t+\alpha \sin\frac{\pi }{T_{s}}t\right]+\Delta_{f}$$

#### 2.1.2. Participants and Experimental Protocol

**Figure 2:**
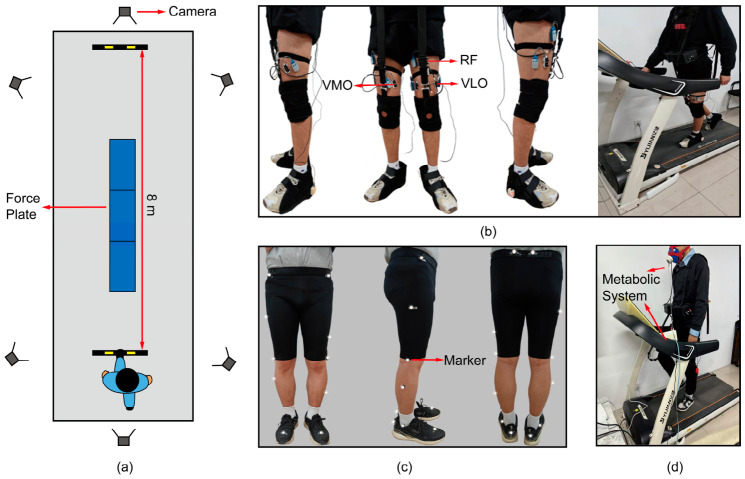
Experimental setup: (**a**) biomechanical data collection; (**b**) surface electromyography data collection; (**c**) plug-in gait marker set; (**d**) metabolic test.

Thirteen healthy participants (8 males, 5 females; 28.08 ± 3.12 years, 1.74 ± 0.09 m, 69.78 ± 8.56 kg) were recruited, consistent with the sample sizes of comparable studies [23,24]. Data acquisition was divided into two parts: biomechanical and sEMG data collection. Detailed subject information is provided in Table A1. The overall experimental setup is illustrated in Figure 2.

The detailed protocol was as follows:

Familiarization: Participants observed a full experiment demonstration, gave informed consent, and completed a lower-limb maximum voluntary contraction (MVC) test guided by a physical therapist.

Biomechanical data collection: Participants performed 20 overground walking trials at a self-selected speed. Kinematic data (100 Hz, VICON, Oxford, UK) and ground reaction forces (1000 Hz, AMTI, Watertown, MA, USA) were synchronously recorded using a standard Plug-in Gait marker set.

sEMG Collection During Gradient Assistance: All participants completed six 1 min gradient-assisted treadmill walking trials at assistance amplitudes of 10, 20, 30, 40, 50, and 60 N (5 min rest between trials). sEMG was recorded (2000 Hz, Noraxon, Scottsdale, AZ, USA) via the myoRESEARCH software (Version 3.20, Noraxon, Scottsdale, AZ, USA) with standard Noraxon bipolar surface electrodes with a fixed inter-electrode distance. Electrode placement was performed by a licensed physical therapist according to SENIAM anatomical guidelines, with standardized skin preparation (shaving and alcohol cleansing) performed prior to attachment to minimize impedance and placement-related variability. Additionally, the Stratified Safety Validation group (SSV, n = 3), selected based on their PDFM-estimated hip flexion moment outputs (spanning low, median, and high biomechanical demands), underwent testing at 70 N and 80 N.

Exoskeleton Assistance Evaluation: Participants completed four conditions: (1) No-exo (NE), (2) Non-assisted (NA), (3) Applied conventional profile (CP), and (4) Applied optimized profile (OP). Each condition included three trials. sEMG was recorded during 1 min walks (5 min rests). Metabolic data were collected for each condition during a protocol of 3 min quiet standing, 6 min walking, and 5 min rest, using a portable system (K4b^2^, COSMED, Rome, Italy). All sessions were supervised by a physical therapist.

Kinematic data (joint coordinates, angles, angular velocities, angular accelerations, and segmental center-of-mass coordinates/accelerations) were exported from Vicon Nexus (Version 2.10.2, VICON, Oxford, UK). Reference hip joint moments were obtained using the Plug-in Gait model [25]. Raw EMG signals were extracted from the middle portion of each trial following adaptation to the testing environment. These signals were band-pass filtered (20–500 Hz, Butterworth), then demeaned, rectified, and low-pass filtered (10 Hz, Butterworth) using myoRESEARCH (Version MR3.20, Noraxon, Scottsdale, AZ, USA), in accordance with Surface EMG for Non-Invasive Assessment of Muscle guidelines. Subsequently, five gait cycles meeting quality criteria were selected and averaged for iEMG calculation to minimize transient artifacts. All subsequent data processing, including gait parameter extraction, EMG analysis, Bayesian optimization, and assistance profile generation, was performed using custom-written scripts in MATLAB (Version R2023a, MathWorks, Natick, MA, USA). This study was approved by the Ethics Committee of Hebei University of Technology (No. [HEBUThMEC2022005]) and conducted in accordance with the Declaration of Helsinki.

### 2.2. Physics-Guided Dynamic Fusion Model (PDFM)

**Figure 3:**
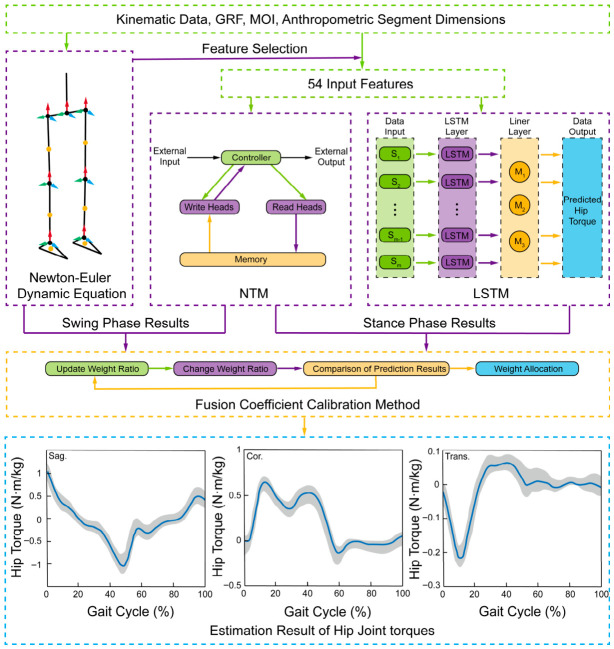
Framework for PDFM. In the Newton–Euler dynamic model, yellow dots denote the segmental centers of mass. The red, green, and blue arrows represent the orthogonal axes of the joint coordinate systems: the red arrow indicates the Z-axis (pointing superiorly/vertically upward), the green arrow indicates the X-axis (pointing to the right), and the blue arrow indicates the Y-axis (pointing anteriorly/forward in the direction of motion).

The Newton–Euler method provides physically generalizable and accurate lower-limb joint moment estimates adaptable to various subjects, yet its rigid reliance on GRF measurements limits its wearable use. Neural network predictors avoid GRFs but suffer from subject variability and reduced accuracy. To overcome these constraints, we designed the PDFM (Figure 3) to improve estimation accuracy without requiring GRF data.

#### 2.2.1. Newton–Euler Inverse Dynamics Model

A simplified seven-link lower limb model (Figure 3) was developed for wearable exoskeleton applications. The model includes the trunk, thighs, shanks, and feet (seven segments with mass centers) and six joints (hips, knees, ankles). Initial segment dimensions were scaled from the anthropometric means of six participants and individually adjusted per user. The hip joint moment from the Newton–Euler inverse dynamics [26] is expressed as follows:

(2) $$M=M(\theta )+F_{m}\times R+I\alpha +\omega \times (I\omega )$$

where *M* is the hip joint moment, *M*(*θ*) represents the projection of other joint moments onto the hip, and *θ* denotes the joint angles. *F*_m_ is the joint force matrix, calculated as the product of segment mass and its center of mass acceleration. *R* is the moment arm, defined as the distance from a segment’s center of mass to its proximal joint. *I* is the moment of inertia, derived from standard segmental formulas, and *α* is the angular acceleration. As indicated by the equation, the input parameters required to drive the Newton–Euler inverse dynamics model include joint coordinates, joint angles, angular velocities, angular accelerations, center of mass coordinates, and their corresponding accelerations. To enhance the neural network model’s capacity to capture biomechanical features, these kinematic parameters were used as input features, totaling 54 dimensions.

#### 2.2.2. Selection of Neural Network Models

An overly complex model architecture can compromise estimation efficiency. Therefore, we adopted a two-factor fusion scheme. To identify the most suitable factors that balance efficiency with accuracy, we first evaluated five distinct neural network architectures for hip joint moment estimation: Backpropagation Neural Network (BPNN), Feedforward Neural Network (FNN), LSTM, NTM, and Recurrent Neural Network (RNN). Leave-one-subject-out (LOSO) cross-validation was employed to validate the estimation accuracy of each neural network approach while ensuring cross-subject generalizability of the models. In this 13-fold procedure, data from twelve participants were used for model training in each iteration, while the remaining participant served as the independent test set. Repeated-measures analysis of variance (ANOVA, see Section 2.4) of the five single neural networks’ rRMSE and correlation coefficients revealed significant performance differences across planes (*p* < 0.001, descriptive statistics in Table 2). Based on their superior overall accuracy, the LSTM and NTM were selected as fusion factors. Detailed statistical comparisons are presented in Section 3.1. Detailed neural network model settings are provided in Table A2.

#### 2.2.3. Construction of the PDFM

The PDFM integrates the LSTM, NTM neural network models, and the Newton–Euler dynamic model. Its workflow is illustrated in Figure 3. During the stance phase, the Newton–Euler model produces erroneous results due to the absence of GRF inputs. Consequently, the fusion is restricted to the LSTM and NTM outputs. In contrast, during the swing phase, the high-performing Newton–Euler model is selected as one factor, paired with the relatively more accurate NTM model as the other.

Preliminary analysis of the five neural networks revealed considerable variation in the estimation accuracy of each individual network across different gait phases. The stance phase constitutes a significant portion of the gait cycle and, crucially, operates entirely without the physics-based guidance of the Newton–Euler method during moment estimation, necessitating a more meticulous approach. Therefore, the stance phase was further subdivided into five distinct stages (Initial contact, Loading response, Mid-stance, Terminal stance, and Pre-swing) for individualized moment estimation.

To rationally orchestrate the fusion of the three sub-models, we designed a Fusion Coefficient Calibration Method (FCCM). The FCCM employs an exhaustive grid search strategy to determine the optimal weight pair for the two selected fusion factors at each gait stage. Specifically, weight coefficients ranging from 0 to 1 (with a step size of 0.01) are independently assigned to each fusion factor, generating a comprehensive set of candidate allocations. The fused output for a given stage is formulated as follows:

(3) $$\tau_{s}=\sum_{p=1}^{6}(w_{p}\tau_{p}^{1}+k_{p}\tau_{p}^{2})$$

where *τ*_s_ is the final hip joint moment estimate output by the PDFM for a specific gait stage, *p* denotes the different gait stages, *τ_p_*^1^ and *τ_p_*^2^ are the estimates from the two sub-models selected for fusion in stage *p*, *w_p_*, and *k_p_* are the weights assigned by FCCM for that stage.

However, determining the optimal weight pair from these candidates requires a unified evaluation criterion. The correlation coefficient quantifies waveform similarity, while root mean square error (RMSE) and rRMSE quantify magnitude accuracy. Relying exclusively on either metric risks neglecting the other dimension of estimation quality. A high correlation coefficient does not preclude substantial amplitude errors. A low rRMSE does not ensure phase fidelity. To integrate these complementary aspects into a single measure, we defined an internal composite objective function termed Correlation-Accuracy (R):

(4) $$R=c\times (1-E_{r})$$

where *c* represents the Correlation Coefficient, and *E*_r_ signifies the rRMSE. The multiplicative form ensures that high *R* can only be achieved when both waveform similarity and magnitude precision are satisfied simultaneously. This metric is used exclusively for FCCM weight calibration and is not employed for final performance evaluation. After evaluating all possible combinations, the specific weight pair (*w_p_*, *k_p_*) yielding the highest value is selected as the optimal allocation for that gait stage. This method not only enhances the overall estimation accuracy but also elucidates the performance characteristics of different algorithms across various gait stages.

### 2.3. Assistance Profile Planning and Optimization

**Figure 4:**
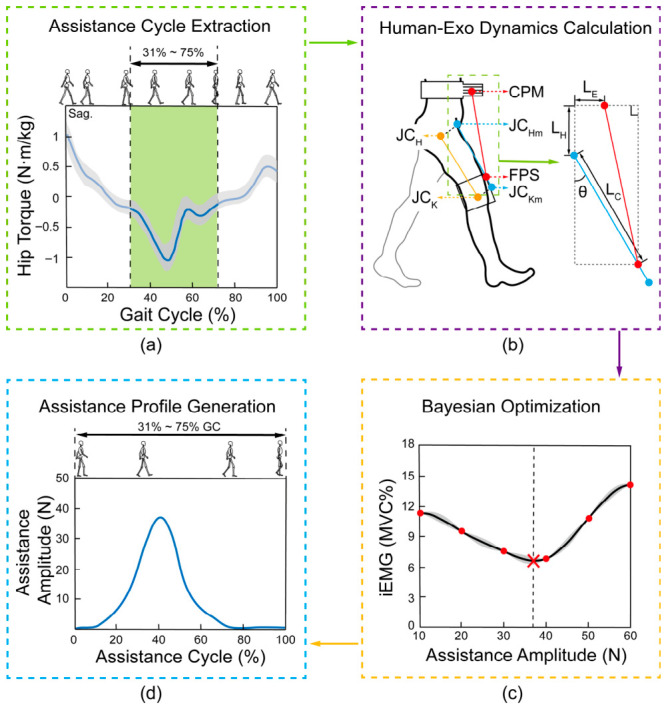
Schematic overview of the assistance profile planning workflow. (**a**) Assistance cycle extraction: the PDFM-estimated sagittal-plane hip flexion moment defines the assistance phase (31–75% GC, green shaded region) spanning terminal stance through mid-swing. (**b**) Human–exoskeleton dynamics calculation: the target hip moment is converted into the required assistance force via the geometric model. The red line represents the projection of the assistive band, connecting its origin and endpoint in the sagittal plane. The yellow line connects the hip and knee joint centers in the sagittal plane. The blue line is a parallel translation of the yellow line within the sagittal plane. The purpose of this translation is to establish an intuitive geometric relationship for computational purposes. (**c**) Bayesian optimization: the optimal assistance amplitude is determined by minimizing the predicted neuromuscular cost (red cross denotes the predicted minimum, and the dashed line marks the corresponding position of the optimized assistance value on the horizontal axis). (**d**) Assistance profile generation: the theoretical force trajectory is parameterized by a 5th-order sum-of-sines model. Panels (**c**,**d**) are schematic illustrations; quantitative results are presented in Figure 5, Figure 6 and Figure A1.

The workflow for assistance profile planning is illustrated in Figure 4. This figure provides a schematic overview of the four sequential steps: (a) assistance cycle extraction and (b) human–exoskeleton dynamics calculation, both detailed in Section 2.3.1; (c) Bayesian optimization of the assistance amplitude, detailed in Section 2.3.2; and (d) final assistance profile generation.

**Figure 5:**
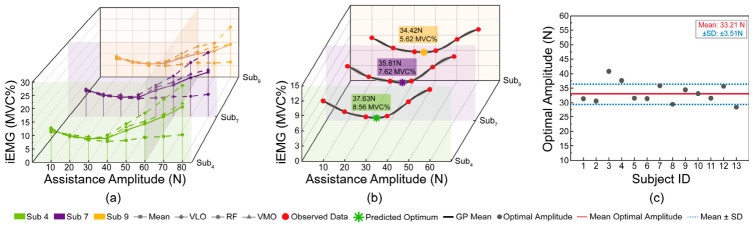
Bayesian optimization of assistance amplitude. (**a**) Mean iEMG (%MVC) of the RF, VLO, and VMO muscles across varying assistance amplitudes for Stratified Safety Validation (SSV) group; (**b**) Gaussian process (GP) regression fitting (solid lines) with observed data points (red circles) and predicted optimal amplitudes (green stars); (**c**) Distribution of individually optimized assistance amplitudes across all 13 subjects, with the mean (red solid line) and standard deviation (blue dashed lines) indicated.

**Figure 6:**
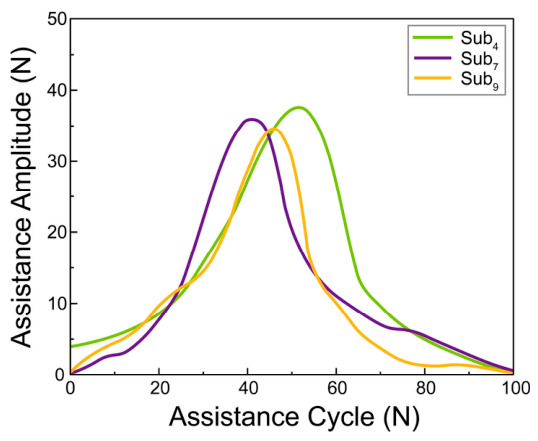
Individualized hip flexion assistance torque amplitude profiles across a full normalized assistance cycle (0–100%) for the SSV group.

#### 2.3.1. Biomechanical Conversion and Profile Generation

Assistance was targeted at the leg-lifting phase (approx. 31–75% of the gait cycle, encompassing terminal stance through mid-swing), as shown in Figure 4a. This interval was determined directly from the sagittal-plane hip flexion moment trajectory estimated by the PDFM, corresponding to the biological period when the hip flexors (primarily the rectus femoris) must generate concentric power to lift the swing leg against gravity. Gait events within this phase were detected using the method from Xiang et al. [27].

With the assistance phase identified, the required exoskeleton force was determined by relating the target hip moment to the mechanical configuration of the human–exoskeleton system. The sagittal-plane dynamic model illustrated in Figure 4b establishes this relationship through the geometry of the assistive band and the lower limb, giving the mapping formula:

(5) $$F=\frac{\tau_{s}}{L_{C}\sin\left[\theta -k\arccos\left(\frac{L_{C}\cos\theta +L_{H}}{\sqrt{{\left(L_{C}\sin\theta -L_{E}\right)}^{2}+{\left(L_{C}\cos\theta +L_{H}\right)}^{2}}}\right)\right]}$$

where *τ*_s_ is the PDFM-derived hip joint moment (the target assistive moment), *F* is the corresponding required assistance force from the exoskeleton, *L*_H_ is the vertical distance in the sagittal plane between the hip joint center and the actuator, *L*_E_ is the sagittal-plane distance from the band attachment point on the exoskeleton to the corresponding point on the wearer’s body, *L*_C_ is the distance in the sagittal plane from the translated hip joint point (blue line) to the band’s endpoint, and *θ* is the hip joint flexion/extension angle. The parameter *k* takes a value of −1 when (*L*_C_ sin*θ* − *L*_E_) ≤ 0, and a value of 1 when (*L*_C_ sin*θ* − *L*_E_) > 0. In this formulation, *τ*_s_ and *θ* are time-varying inputs. The remaining parameters are anthropometrically determined constants for a given user and setup. Therefore, *F* can be expressed as a time-varying function *f(t)* and is presented in the form of an assistance force profile.

While the theoretical force distribution curve *f(t)* can be directly adopted as the reference basis for the design of the assistive function, its multiple input parameters and computational complexity would impede the exoskeleton’s response speed. Therefore, a sum-of-sines model was adopted to generate the exoskeleton’s assistance function *F(t)* [28], expressed as:

(6) $$F(t)=\sum_{m=1}^{n}a_{m}\sin(b_{m}t+c_{m})$$

where *n* denotes the order (number of harmonic terms) of the function, and *a_m_*, *b_m_*, *c_m_* are the amplitude, frequency, and phase parameters, respectively. These parameters were determined by fitting the model to the target profile *f(t)* using nonlinear least-squares optimization with a trust-region iterative strategy, minimizing the sum of squared residuals.

Given that the sum-of-sines model involves a boundary constraint related to the assigned order *n*, assistance functions from the 1st to the 8th order (*n* = 1, 2, …, 8) were constructed. The rRMSE and R^2^ between each order’s function and *f(t)* were calculated, as summarized in Table 1.

Results indicate that accuracy did not improve significantly beyond the 5th order. Consequently, a 5th-order function was selected as the final assistance profile. Because the raw force magnitude from rigid-body modeling was found mechanically unreliable, the assistance function for the i-th participant was normalized into the following form:

(7) $$f_{i}(t)=A_{i}\frac{f(t)}{f_{i\max}}=A_{i}\frac{\sum_{m=1}^{5}a_{m}\sin(b_{m}t+c_{m})}{f_{i\max}}$$

where *A_i_* is the assistance amplitude for the exoskeleton applied to the i-th participant, and *f_i_*_max_ is the maximum value of the original target profile *f_i_(t)*.

#### 2.3.2. Bayesian Optimization and Personalized Calibration

With the assistance waveform defined, this section optimizes its amplitude. Previous studies typically set a fixed amplitude (e.g., 35 N) empirically, requiring per-user field adjustment. To overcome this, we propose a muscle-activity-based customization method. As a prerequisite, a physiologically safe assistance range needs to be established.

Consistent with biological torque control principles [10], the assistance force required for effective load sharing scales with the biological hip flexion moment demand [29]. Accordingly, three participants were selected to constitute the Stratified Safety Validation group based on their PDFM-estimated hip flexion moment outputs during unassisted walking, with normalized peak hip flexion moments spanning the lower quartile (< 25th percentile, sub_9_), median (~50th percentile, sub_7_), and upper quartile (>90th percentile, sub_4_), respectively. After generating their waveform *f*_SSV_*(t)*, *sub*_SSV_ underwent comprehensive gradient testing at nine assistance levels (10–80 N, with 80 N being the motor output limit, in 10 N increments). sEMG of RF, VMO, and VLO was recorded, and iEMG was calculated over five assistance cycles (31–75% gait cycle) and normalized as follows [30]:

(8) $${\mathrm{iEMG}}_{\mathrm{SSV}}^{K}(\mathrm{MVC}\%)=\left(\sum_{n=1}^{5}\frac{iEMG_{\mathrm{SSV},n}^{K}}{iEMG_{\mathrm{SSV}}^{\mathrm{MVC}}}\times 100\%\right)/5$$

where *K* = 10, 20, …, 80, and SSV denotes Subjects 4, 7, and 9. The results are presented in Figure 5a. Within the 10–40 N range, muscle activity levels decreased steadily, indicating effective load sharing by the exoskeleton. However, when the assistance amplitude exceeded 60 N, the iEMG of the VMO and VLO increased sharply. This is because an excessively high assistive force, mismatched with the natural gait dynamics, induced an overly rapid hip flexion velocity. Consequently, increased knee extension velocity was required to maintain gait stability. Furthermore, the rise in iEMG for the RF within this high-assistance range reflects abnormal compensatory activity. Therefore, the optimal assistance amplitude was set at 60 N in this study.

To identify the personalized optimal assistance amplitude that minimizes muscle activation, we employed a Gaussian Process (GP)-based Bayesian optimization approach. Given the discrete nature of empirical sampling, six assistance levels (10, 20, 30, 40, 50, and 60 N) were tested for each participant, yielding corresponding mean iEMG values (averaged across RF, VMO, and VLO). These six observations served as training data for a GP surrogate model with a Matérn 5/2 kernel, which was fitted to characterize the probabilistic relationship between assistance amplitude and muscle activation. The GP provides a posterior predictive distribution comprising a mean function *μ(A)* (predicted iEMG) and standard deviation *σ(A)* (epistemic uncertainty), enabling interpolation across the continuous search space 10–60 N. The optimal amplitude was determined by minimizing the GP posterior mean within the SSV-validated safety bounds [31]:

(9) $$A_{i}^{*}=\arg\underset{A\in [10,60]}{\min}\mu (A)$$

For this one-dimensional problem with six uniformly distributed observations, minimizing the GP posterior mean yields the converged Bayesian optimization solution while leveraging the GP’s capacity for uncertainty quantification. From a biomechanical perspective, this optimization aims to identify the assistance amplitude that effectively reduces the load on the RF without inducing compensatory activation in the VMO and VLO, thereby minimizing the overall neuromuscular cost.

**Figure A1:**
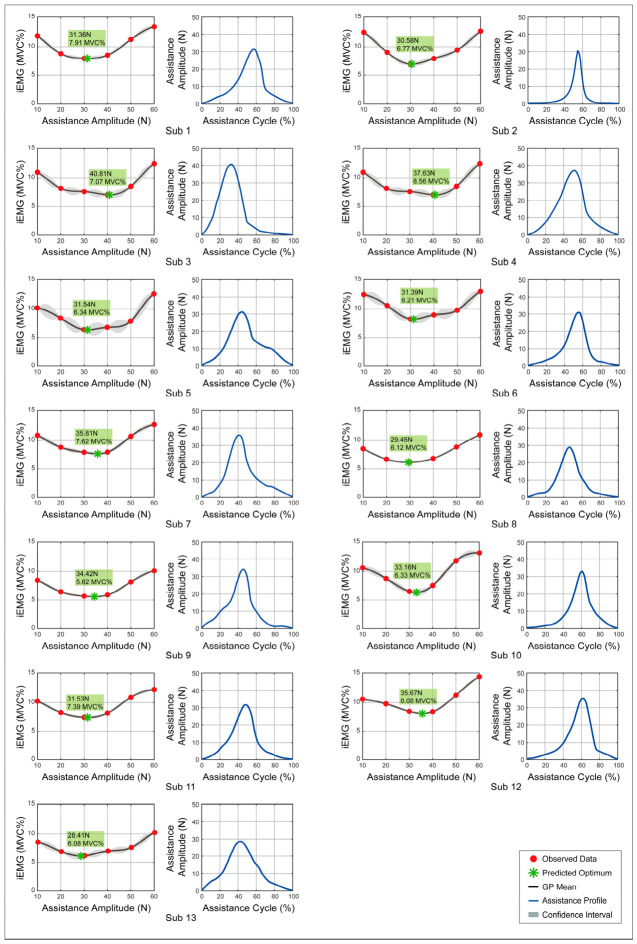
Complete Bayesian optimization results and generated assistance profiles for all 13 subjects.

Representative Bayesian optimization curves for the SSV group are presented in Figure 5b, illustrating the GP posterior mean, 95% confidence intervals, and predicted optima across low, moderate, and high biomechanical demand profiles. The distribution of resulting optimal amplitudes for all participants is shown in Figure 5c. Finally, the personalized assistance profiles (time-domain force curves) generated from these optimized amplitudes are illustrated in Figure 6. Complete Bayesian optimization results and generated assistance profiles for all 13 subjects are presented in Figure A1.

### 2.4. Statistical Analysis

All statistical analyses were performed using IBM SPSS Statistics (Version 26.0, IBM Corp., Armonk, NY, USA), with a two-tailed significance level set at α = 0.05, and all data presented as mean ± SD. Prior to hypothesis testing, the Shapiro–Wilk test was used to verify the normality of data distribution, and Mauchly’s test was applied to validate the sphericity assumption, with Greenhouse–Geisser correction employed to adjust degrees of freedom when sphericity was violated. One-way repeated-measures analysis of variance (ANOVA) was conducted to evaluate differences in multi-plane hip joint moment estimation accuracy across 7 models (BPNN, FNN, LSTM, NTM, RNN, N–E, PDFM) and differences in neuromuscular/metabolic outcomes across 4 experimental conditions, with Bonferroni-corrected post hoc tests used for pairwise comparisons to control Type I error, and partial eta squared (${\eta_{p}}^{2}$) reported as the effect size to quantify the magnitude of observed effects, with 0.01, 0.06 and 0.14 defined as small, medium and large effects per Cohen’s standard.

## 3. Results

### 3.1. Accuracy of Hip Moment Output by PDFM

**Figure 7:**
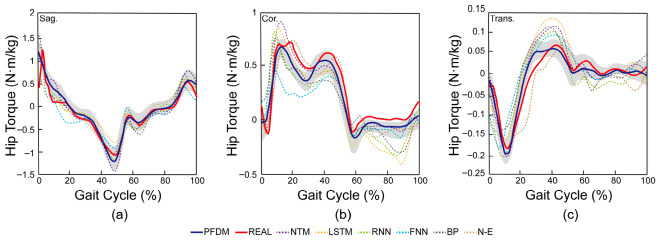
Comparison of hip joint moment trajectories in (**a**) sagittal, (**b**) coronal, and (**c**) transverse planes. Solid blue line represents PDFM mean across thirteen subjects with shaded area indicating ±1 standard deviation; solid red line represents ground truth (Vicon-based inverse dynamics); dotted lines represent single neural network baselines (NTM, LSTM, RNN, FNN, BPNN) and Newton–Euler model shown as mean trajectories without error bands for clarity. The standard deviations are illustrated by the shaded area.

To demonstrate the superior performance of the proposed PDFM, LOSO cross-validation was conducted for the model. The leave-one-subject-out (LOSO) cross-validation results (n = 13) are presented in Figure 7 and Table 2.

First, to select fusion factors from the five standalone neural networks, we evaluated their hip joint moment prediction performance using ANOVA. Significant main effects of network type were found for both the correlation coefficient and rRMSE across all three planes (*p* < 0.001). Post hoc comparisons indicated that although LSTM (0.879 ± 0.036) and NTM (0.889 ± 0.035) exhibited lower correlation coefficients than RNN (0.891 ± 0.034) in the sagittal plane (*p* < 0.01), they significantly outperformed the other three models in the coronal and transverse planes (*p* < 0.001). For rRMSE, LSTM and NTM were also significantly lower than the other three models in the coronal and transverse planes (*p* < 0.05). Notably, LSTM recorded the lowest sagittal-plane rRMSE (6.30 ± 2.17%), while NTM showed a relatively higher value (10.83 ± 2.65%). Given that LSTM and NTM demonstrated superior performance over the other models in the majority of evaluated metrics and planes, which reflected their complementary plane-specific strengths, they were selected as the fusion factors.

Subsequently, we employed ANOVA to assess the performance of all seven models. For correlation coefficients, which evaluate temporal similarity and phase consistency, significant main effects were observed across all three planes: in the sagittal plane, *F* (1.03, 12.38) = 319.959, *p* < 0.001, ${\eta_{p}}^{2}$ = 0.964; in the coronal plane, *F* (1.13, 13.57) = 309.512, *p* < 0.001, ${\eta_{p}}^{2}$ = 0.963; and in the transverse plane, *F* (1.04, 12.43) = 160.598, *p* < 0.001, ${\eta_{p}}^{2}$ = 0.930. Post hoc comparisons indicated that PDFM achieved significantly higher correlation coefficients than all comparative models in the sagittal (0.938 ± 0.028, *p* < 0.001) and transverse (0.929 ± 0.029, *p* < 0.001) planes. However, in the coronal plane, while PDFM (0.924 ± 0.024) significantly exceeded the five single neural networks (all *p* < 0.001), it was significantly outperformed by the Newton–Euler model (0.939 ± 0.022, *p* < 0.001).

Regarding rRMSE, significant model effects were detected: in the sagittal plane, *F* (6,72) = 67.665, *p* < 0.001, ${\eta_{p}}^{2}$ = 0.849; in the coronal plane, *F* (6,72) = 21.280, *p* < 0.001, ${\eta_{p}}^{2}$ = 0.639; and in the transverse plane, *F* (6,72) = 82.287, *p* < 0.001, ${\eta_{p}}^{2}$ = 0.873. PDFM exhibited significantly lower rRMSE than all other models in the sagittal (5.29 ± 1.79%, all *p* < 0.01) and transverse (5.61 ± 5.18%; all *p* < 0.001) planes. However, in the coronal plane, PDFM (9.79 ± 3.14%) exhibited significantly lower rRMSE than all single neural networks (all *p* < 0.01) but was significantly higher than the Newton–Euler model (7.83 ± 2.02%, *p* = 0.004). Consistent trends were observed for absolute RMSE.

These results demonstrate the overall predictive superiority of PDFM over single-model baselines, validating the effectiveness of the fusion strategy in reducing GRF dependency for wearable applications. Furthermore, descriptive statistics in Table 2 indicate that for most standalone models, sagittal-plane estimation outperformed coronal and transverse planes. This discrepancy originates from the inherent biomechanical characteristics of the ground truth: sagittal-plane motion exhibits relatively stable and pronounced patterns, whereas coronal and transverse moments demonstrate higher inter-subject variability. In contrast, PDFM maintained high estimation accuracy across all three planes, with its most pronounced advantages observed in the coronal and transverse planes. This can likely be attributed to the stability and complementarity of the three constituent models dynamically allocated by FCCM in these planes. Notably, in the coronal plane—where muscle torque contributions are relatively limited—the Newton–Euler physics-based model, grounded in inverse dynamics, exhibited high predictive performance, contributing to the fusion model’s competitive coronal-plane accuracy.

Table 3 presents the dynamic weight allocations by the FCCM across gait phases. During the stance phase, NTM was generally assigned higher weights across most phases and planes. However, its weight was lower than that of LSTM in specific instances: the sagittal plane during Loading Response, the coronal plane during Midstance, and both the transverse and coronal planes during Terminal Stance. In contrast, during the swing phase, NTM’s weight exceeded that of the Newton–Euler model only in the transverse plane. These heterogeneous allocations suggest that model superiority is gait-phase-dependent, and the FCCM effectively exploits the complementary strengths of each sub-model rather than relying on a static fusion strategy.

### 3.2. Bayesian Optimization Outcomes

The Bayesian optimization identified individually optimal assistance amplitudes that minimized the predicted neuromuscular cost for each participant. As illustrated in Figure 5c, the optimized amplitudes ranged from 28.41 N to 40.81 N (mean: 33.21 ± 3.51 N). Although the cohort mean approximated the empirically fixed value of 35 N, the individual distribution exhibited marked deviation from this uniform target: 3 of 13 subjects (23.08%) converged to optimal amplitudes >40 N or <30 N. This non-negligible inter-subject variability underscores the necessity of subject-specific calibration and suggests that a fixed-profile approach would fail to accommodate a substantial subset of users.

### 3.3. Electromyographic and Metabolic Effect of the Assistance

iEMG serves as a surrogate measure of neural drive; reductions under submaximal contractions are associated with decreased efferent electrical signals and motor unit recruitment. Therefore, we employed iEMG to evaluate the efficacy of the optimized assistance profile.

**Figure 8:**
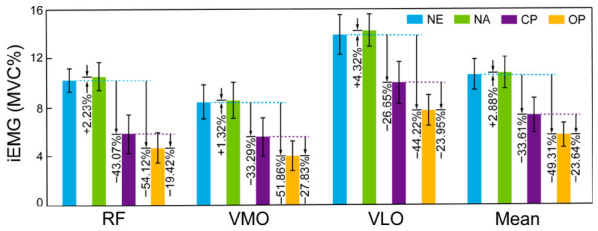
Muscle activation levels (iEMG, %MVC) of the RF, VMO, and VLO during walking under four experimental conditions: no-exo (NE), non-assisted (NA), Applied conventional profile (CP), and Applied optimized profile (OP). Error bars indicate standard deviations. Percentage values positioned to the left of each bar represent changes relative to the NE condition, whereas values to the right represent changes relative to the CP condition.

The iEMG of the three target muscles and their mean values were compared across four testing conditions (Figure 8 and Table 4): NE, NA, CP, and OP. ANOVA revealed significant differences in mean iEMG across conditions: *F* (1.00, 13.00) = 4332.586, *p* < 0.001, ${\eta_{p}}^{2}$ = 0.997. Compared to the NE condition (10.77 ± 1.19%MVC), mean iEMG was significantly reduced by 49.31% in OP (5.46 ± 1.01%MVC) and 33.61% in CP (7.15 ± 1.37%MVC) (*p* < 0.001), with the RF showing the greatest reduction among the three target muscles (54.12% and 43.07%, respectively). This reduction is consistent with the successful unloading of the target muscles by the exosuit assistance. Compared to CP, the mean iEMG decreased by 23.64% in OP.

Relative iEMG reductions exhibited different patterns across baselines. Compared with NE, OP showed a significantly larger reduction in RF (54.12%) than in VLO (44.22%) and VMO (51.86%) (all *p* < 0.03). In contrast, compared with CP, the reduction in RF (19.42%) was significantly smaller than those in VLO (23.95%) and VMO (27.83%) (all *p* < 0.001). This differential pattern suggests that the optimized assistance achieved more selective unloading of the primary target muscle (RF) while minimizing concurrent activation demands on synergistic knee extensors (VLO and VMO), whereas the conventional profile appeared to distribute mechanical assistance less discriminately across the muscle group.

**Figure 9:**
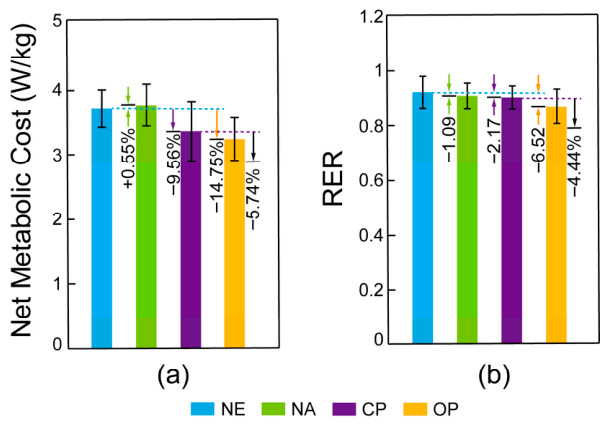
(**a**) Net metabolic cost (W/kg) and (**b**) respiratory exchange ratio (RER) across four experimental conditions: no-exo (NE), non-assisted (NA), Applied conventional profile (CP), and Applied optimized profile (OP). Error bars indicate standard deviations. Percentage values positioned to the left of each bar represent changes relative to the NE condition, whereas values to the right represent changes relative to the CP condition.

To determine whether the observed neuromuscular benefits translated into systemic metabolic savings, we next examined whole-body energy expenditure and substrate utilization. Both metabolic cost and respiratory exchange ratio (RER) differed significantly across the four conditions: metabolic cost, *F* (1.05, 12.60) = 200.416, *p* < 0.001, ${\eta_{p}}^{2}$ = 0.944; RER, *F* (1.08, 12.91) = 10.741, *p* < 0.001, ${\eta_{p}}^{2}$ = 0.472 (Figure 9 and Table 4). Compared to the NE condition (3.66 ± 0.28 W/kg), net metabolic cost significantly decreased in OP (3.12 ± 0.36 W/kg) and CP (3.31 ± 0.44 W/kg) by 14.75% and 9.56%, respectively (all *p* < 0.001). Furthermore, RER in the OP condition (0.86 ± 0.06) was significantly lower than in NE (0.92 ± 0.05), NA (0.91 ± 0.04), and CP (0.90 ± 0.04) (all *p* < 0.001), consistent with improved metabolic efficiency. Restricted to CP and OP conditions, ANOVA indicated significantly reduced metabolic cost and RER in the optimized profile: *F* (1,12) = 82.273, *p* < 0.001, ${\eta_{p}}^{2}$ = 0.873 for metabolic cost; and *F* (1,12) = 51.896, *p* < 0.001, ${\eta_{p}}^{2}$ = 0.812 for RER, suggesting the effectiveness of the profile optimization. The concurrent reduction in both metabolic cost and RER is consistent with the interpretation that optimized assistance may not only reduce gross energy expenditure but also reflect a shift toward more efficient substrate utilization, potentially indicative of decreased physiological strain.

## 4. Discussion

This study developed a high-accuracy hip joint moment estimation model (PDFM) that eliminates dependence on ground reaction forces and customized the exoskeleton assistance profile based on its output to enhance assistive efficacy. The results demonstrate that PDFM not only exhibits high biomechanical interpretability but also surpasses traditional biomechanical and single-network computational methods in hip joint moment estimation accuracy. In this healthy young adult cohort, the optimized assistance profile significantly reduced the load on the primary target muscles and the overall metabolic cost while improving muscle coordination patterns. Whether these neuromuscular benefits translate similarly to populations with altered biomechanics (e.g., gait asymmetry, muscle weakness) requires dedicated investigation.

### 4.1. Performance and Biomechanical Interpretability of the PDFM

The PDFM achieved the highest accuracy in estimating hip joint moments across all three anatomical planes, outperforming all single neural network baselines and thereby validating the effectiveness of its complementary learning fusion strategy. Notably, the accuracy improvements provided by PDFM in the coronal and transverse planes were substantially greater than those in the sagittal plane. This observation aligns with established biomechanical characteristics of gait: during level walking, sagittal-plane motion exhibits relatively stable and pronounced patterns, whereas coronal and transverse moments demonstrate higher inter-subject variability and rely more heavily on whole-body coordination to maintain dynamic balance [32]. It is noteworthy, however, that PDFM did not surpass the Newton–Euler model in the coronal plane, where hip moments are predominantly generated by the GRF acting through a relatively constant frontal-plane lever arm. In this plane, the contribution of muscle torque to the net hip moment is relatively limited. The hip abductor muscles primarily function to stabilize the pelvic center of mass against gravity via force-couple modulation rather than to generate large rotational moments about the hip joint, making the moment waveform closely mirror the GRF magnitude profile [33]. Consequently, the Newton–Euler model, which directly incorporates GRF measurements, retains an inherent informational advantage that kinematics-driven PDFM cannot fully replicate [34]. Nevertheless, PDFM still outperformed all single neural networks in the coronal plane, underscoring the value of physics-guided fusion for non-sagittal estimation.

The performance differences between LSTM and NTM across anatomical planes can likely be attributed to the correspondence between the inherent biomechanical control characteristics of each plane and the computational strengths of the respective networks. During level walking, sagittal-plane hip moments are predominantly governed by periodic, velocity-dependent propulsion patterns. Although their magnitude varies substantially throughout the gait cycle, the waveforms are continuous and smooth, exhibiting strong temporal autocorrelation. This is precisely the domain where LSTM’s gating mechanisms excel, enabling accurate capture and prediction of such long-range, smooth temporal dynamics [35,36]. In contrast, although coronal and transverse moments are also phase-locked to the gait cycle, their control strategies are dominated by intermittent, event-driven balance corrections and whole-body coordination tasks. These non-periodic, high-variability patterns require rapid template retrieval and matching rather than sequential extrapolation, a capacity that aligns with NTM’s content-addressable external memory architecture, which enables explicit storage and recall of complex, subject-specific motor templates [37]. Consequently, the PDFM fusion strategy achieves performance gains through “architecture-aware biomechanical matching”: LSTM handles the rhythmic propulsion dynamics in the sagittal plane, while NTM manages the complex dynamics in non-sagittal planes that are dominated by discrete, memory-intensive balance-control tasks. This also explains why the fusion gains are most pronounced in the coronal and transverse planes: in these planes, single-network approaches struggle to simultaneously address both temporal dynamic modeling and template memory and thus fail to capture the full complexity of balance-control dynamics.

These architecture-biomechanics correspondences are reflected in the phase-specific weight allocations determined by FCCM (Table 3). During propulsion-oriented sub-phases (Loading Response and Terminal Stance in the sagittal plane), LSTM received higher weights, whereas NTM dominated balance-critical phases (Initial Contact, Midstance, and Preswing) across all planes. Notably, during Swing—when GRF is absent—the Newton–Euler model contributed substantially in the sagittal and coronal planes, reflecting its physical consistency under inertial-dominant conditions. In the transverse plane, however, NTM remained dominant during Swing. This can likely be attributed to the inherent modeling challenges of hip rotational dynamics [38], where the small moment of inertia about the longitudinal axis renders the motion highly sensitive to soft-tissue artifacts and individual-specific anatomical variations in femoral torsion. Under these conditions, the Newton–Euler model’s rigid-body assumptions and standard segmental inertial parameters introduce non-negligible simplification errors, whereas NTM’s memory-based template matching can implicitly capture these nuanced rotational patterns directly from kinematic data without relying on precise inertial parameterization.

The PDFM demonstrates technical potential for wearable applications in principle, as its input features (joint kinematics) are compatible with IMU-based motion capture systems, though the current validation relied on laboratory-grade optical tracking; whether IMU-derived kinematics can achieve comparable PDFM accuracy in unconstrained environments remains to be tested. Crucially, the high-accuracy hip joint moment estimates provided by the PDFM serve as the essential biomechanical input for the human–exoskeleton dynamics model, enabling the generation of physiologically consistent assistance profiles. This precise estimation capability establishes the necessary foundation for subject-specific customization, ensuring that the derived assistance parameters align with individual biomechanical demands.

### 4.2. Necessity of Subject-Specific Assistance Amplitude Calibration

While the PDFM-derived assistance profile provides a biomechanically consistent waveform, its amplitude requires subject-specific calibration to match individual load-sharing demands. The considerable inter-subject variability in optimal assistance amplitudes (28.41–40.81 N) reflects heterogeneity in individual biomechanical demand rather than optimization noise. The complete Bayesian optimization results for all 13 subjects (Figure 5c) demarcate the practical boundaries of personalized assistance: the upper extreme (Sub3, 40.81 N) suggests that high-demand individuals require substantially greater support than the empirical 35 N to achieve effective load sharing, whereas the lower extreme (Sub13, 28.41 N) indicates that low-demand individuals may experience suboptimal or destabilizing over-assistance under a fixed profile (Figure A1). These findings underscore that a fixed 35 N profile is biomechanically invalid for a substantial subset of users. In contrast, the PDFM-based moment estimation enables subject-specific amplitude scaling that respects individual biomechanical demand boundaries.

### 4.3. Neuromuscular and Metabolic Implications

The assistance profile optimized based on PDFM output and iEMG-guided Bayesian optimization exhibited statistically significant improvements across the evaluated neuromuscular and metabolic parameters. At the muscle activity level, OP reduced the overall iEMG of the target muscle group by 49.31% compared to NE, consistent with effective load sharing by the exoskeleton. Changes in muscle activation patterns were observed between conditions. When transitioning from the NE condition to the OP condition, the primary reduction in activation occurred in the RF. In contrast, when shifting from the CP condition to the OP condition, greater reductions were observed in the VLO and VMO relative to the RF. This suggests that the CP condition may have elicited different recruitment strategies among the quadriceps components, whereas the optimized profile was associated with a redistribution of mechanical demand favoring the primary hip flexor (RF). Mechanistically, this divergence likely reflects a “knee compensation” effect induced by the conventional profile’s suboptimal assistance timing. Biomechanically, an excessively fixed waveform would likely generate excessive hip flexion velocity during late stance, which, in turn, would likely require increased knee extensor activity (VMO/VLO) to stabilize the lower limb during weight acceptance. In contrast, the optimized profile—derived from PDFM’s subject-specific moment estimates—better synchronized the assistive force with the biological hip flexion moment trajectory, thereby attenuating the elevated knee extension demand and allowing VMO/VLO to operate at reduced activation levels. This redistribution of mechanical demand—from apparent compensatory knee extension to direct hip flexion assistance—is consistent with a shift toward more balanced muscle coordination. These condition-dependent differences in muscle activation distribution are consistent with the enhanced customization achieved by the optimized assistance profile. The observed iEMG reductions were accompanied by concordant decreases in metabolic cost and RER. Because these metabolic and respiratory parameters were assessed through indirect calorimetry independently of the sEMG acquisition chain, a measurement-artifact explanation is unlikely.

The optimization at the muscular level was ultimately reflected in a reduction in whole-body metabolic cost. During the OP condition, net metabolic cost decreased by 14.75% compared to NE and by 5.74% compared to the CP condition. This differential advantage of OP over CP cannot be attributed to mass effects, as the exoskeleton mass (2.7 kg) was identical across all assisted conditions. Within the constraints of this healthy cohort, the 14.75% metabolic reduction relative to no-exo walking is consistent with the effective range reported for recent hip-assistive systems, including single-drive (10.1%) [39], cable-driven (11.35%) [1], dual-disc motor-driven (8.8%) [40], and unpowered (7.2%) [9] devices. The more salient finding, however, is the 5.74% metabolic advantage of OP over CP, which was obtained with identical exoskeleton hardware and mass and thereby isolates the specific contribution of personalization from device-related confounds. This within-device comparison demonstrates that the proposed biomechanically informed optimization framework achieves meaningful metabolic benefits beyond what is attainable through empirical curve tuning alone. Furthermore, the concurrent decrease in RER (from 0.91 to 0.86) is consistent with a modest increase in the relative contribution of fat oxidation to total energy expenditure [41]. This may reflect a reduction in overall physiological load while maintaining the same walking speed, potentially contributing to decreased perceived exertion.

### 4.4. Limitations and Future Work

This study has several limitations. First, the sample comprised healthy young adults (n = 13), which restricts the direct generalization of these findings to older adults or individuals with gait impairments. Second, all experiments were conducted in a controlled laboratory setting with overground walking at self-selected speeds; therefore, the ecological validity and transferability to complex real-world scenarios remain to be established. Third, while PDFM demonstrates high estimation accuracy under offline analysis conditions, its computational latency and integration with low-latency real-time control architectures have not been validated.

Future research will address these limitations through three targeted directions. First, we will prospectively validate the PDFM framework in broader user populations, including older adults and clinical cohorts (e.g., post-stroke hemiparesis, sarcopenia). We explicitly note that the empirical parameters, including FCCM weights and Bayesian optimization response surfaces, were derived exclusively from healthy gait and may require population-specific recalibration. Whether the observed personalization fidelity extends to individuals with higher baseline asymmetry or to those with compensatory gait patterns remains an open empirical question. Second, we will transition the experimental protocol from laboratory settings to real-world environments (e.g., community ambulation, uneven terrain) to evaluate the robustness of PDFM-based assistance under ecologically valid conditions. Third, engineering efforts will focus on optimizing the computational pipeline for edge deployment, enabling closed-loop, real-time moment estimation and assistance adaptation on wearable embedded systems.

## 5. Conclusions

This study proposes a fusion model (PDFM) for high-accuracy hip joint moment estimation. The model achieved correlation coefficients of 0.938, 0.924, and 0.929, and rRMSE values of 5.29%, 9.79%, and 5.61%, in the sagittal, coronal, and transverse planes, respectively. These results outperformed all single-network baselines. Furthermore, the biomechanically interpretable weight allocations (Table 3) elucidate the complementary strengths of physics-based and data-driven approaches. This consistent high-fidelity estimation (correlation coefficient > 0.92 and rRMSE < 10% in all planes), achieved without ground reaction force measurements, establishes a critical foundation for wearable applications. Leveraging the sagittal-plane moment estimates from the PDFM, a customized assistance profile was derived through human-exo dynamic formulation and Bayesian amplitude optimization. In subsequent wearable experiments, this optimized profile reduced target muscle group activity by approximately 49.31%, lowered net metabolic cost by 14.75%, and significantly decreased the RER from 0.92 to 0.86. These results demonstrate that personalized assistance optimization, driven by high-fidelity biomechanical perception (via PDFM), can synergistically achieve significant neuromuscular unloading and metabolic savings. This work provides a validated framework and crucial empirical evidence to inform the development of next-generation intelligent and efficient wearable assistive systems.

## Figures and Tables

**Table 1:** rRMSE and R^2^ of each order of boost function.

Assistance Function Order	1	2	3	4	5	6	7	8
rRMSE	21.8%	7.49%	2.58%	0.53%	0.19%	0.18%	0.18%	0.17%
R^2^	0.554	0.949	0.994	0.999	1	1	1	1

Note: R^2^, coefficient of determination.

**Table 2:** Performance comparison of moment estimation accuracy (Mean ± SD) across three planes.

Model	Correlation Coefficient			rRMSE			RMSE (N·m/kg)		
	Sag.	Cor.	Trans.	Sag.	Cor.	Trans.	Sag.	Cor.	Trans.
BPNN	0.867	0.813	0.817	8.09%	21.73%	19.82%	0.182	0.191	0.045
	±0.046	±0.033	±0.032	±2.95%	±5.51%	±8.21%	±0.072	±0.037	±0.018
FNN	0.871	0.801	0.767	7.37%	21.52%	23.40%	0.163	0.189	0.053
	±0.032	±0.049	±0.072	±3.59%	±6.25%	±8.47%	±0.081	±0.049	±0.019
LSTM	0.879	0.895	0.871	6.30%	19.71%	16.13%	0.141	0.175	0.037
	±0.036	±0.032	±0.033	±2.17%	±4.32%	±6.45%	±0.052	±0.036	±0.016
NTM	0.889	0.904	0.915	10.83%	16.96%	14.12%	0.242	0.149	0.032
	±0.035	±0.031	±0.038	±2.65%	±5.12%	±8.66%	±0.062	±0.033	±0.018
RNN	0.891	0.849	0.787	8.67%	19.81%	18.03%	0.191	0.177	0.041
	±0.034	±0.048	±0.065	±1.01%	±5.88%	±6.72%	±0.048	±0.048	±0.015
N–E	0.935	0.939	0.897	7.89%	7.83%	13.62%	0.175	0.071	0.031
	±0.031	±0.022	±0.032	±1.13%	±2.02%	±5.27%	±0.043	±0.023	±0.012
PDFM	0.938	0.924	0.929	5.29%	9.79%	5.61%	0.117	0.086	0.013
	±0.028	±0.024	±0.029	±1.79%	±3.14%	±5.18%	±0.041	±0.026	±0.012

Note: Sag., sagittal plane; Cor., coronal plane; Trans., transverse plane.

**Table 3:** Weight allocation results of FCCM.

Dimensions	Initial Contact		Loading Response		Mid Stance		Terminal Stance		Pre-Swing		Swing	
	*w* ^LSTM^	*k* ^NTM^	*w* ^LSTM^	*k* ^NTM^	*w* ^LSTM^	*k* ^NTM^	*w* ^LSTM^	*k* ^NTM^	*w* ^LSTM^	*k* ^NTM^	*w* ^N–E^	*k* ^NTM^
Sag.	0.06 ± 0.02	0.71 ± 0.05	0.88 ± 0.09	0.46 ± 0.07	0.29 ± 0.11	0.93 ± 0.14	0.95 ± 0.17	0.44 ± 0.11	0.41 ± 0.06	0.62 ± 0.13	0.89 ± 0.09	0.25 ± 0.08
Cor.	0.22 ± 0.05	0.92 ± 0.17	0.07 ± 0.02	0.74 ± 0.07	0.77 ± 0.08	0.12 ± 0.06	0.85 ± 0.09	0.37 ± 0.04	0.22 ± 0.05	0.91 ± 0.07	0.61 ± 0.12	0.28 ± 0.03
Trans.	0.11 ± 0.04	0.69 ± 0.08	0.05 ± 0.04	0.98 ± 0.16	0.09 ± 0.04	0.85 ± 0.12	0.13 ± 0.05	0.56 ± 0.13	0.07 ± 0.02	0.86 ± 0.06	0.62 ± 0.14	0.87 ± 0.07

Note: Sag., sagittal plane; Cor., coronal plane; Trans., transverse plane.

**Table 4:** Muscle activity and metabolic parameters under different conditions.

Condition	RF (%)	VMO (%)	VLO (%)	Mean (%)	Metabolic Cost (W/kg)	RER
NE	10.31 ± 1.35	8.35 ± 1.42	13.66 ± 1.63	10.77 ± 1.19	3.66 ± 0.28	0.92 ± 0.05
NA	10.54 ± 1.13	8.46 ± 1.49	14.25 ± 1.32	11.08 ± 1.26	3.68 ± 0.32	0.91 ± 0.04
CP	5.87 ± 1.58	5.57 ± 1.58	10.02 ± 1.71	7.15 ± 1.37	3.31 ± 0.44	0.90 ± 0.04
OP	4.73 ± 1.19	4.02 ± 1.22	7.62 ± 1.22	5.46 ± 1.01	3.12 ± 0.36	0.86 ± 0.06

## Data Availability

The original data are available following a reasonable request.

## Abbreviations

The following abbreviations are used in this manuscript:

PDFM	Physics-guided Dynamic Fusion Model
PINN	Physics-informed Neural Networks
N–E	Newton–Euler
EMG	Electromyography
GRF	Ground Reaction Force
IMU	Inertial Measurement Unit
LSTM	Long Short-Term Memory
rRMSE	Relative Root Mean Square Error
NTM	Neural Turing Machines
sEMG	Surface Electromyography
iEMG	Integrated Electromyography
RF	Rectus Femoris
VMO	Vastus Medialis Obliquus
VLO	Vastus Lateralis Obliquus
MVC	Maximum Voluntary Contraction
NE	No-exo
NA	Non-assisted
CP	Applied Conventional Profile
OP	Applied Optimized Profile
BPNN	Backpropagation Neural Network
FNN	Feedforward Neural Network
RNN	Recurrent Neural Network
LOSO	Leave-One-Subject-Out
FCCM	Fusion Coefficient Calibration Method
SSV	Stratified Safety Validation
GP	Gaussian Process
ANOVA	One-Way Repeated-measures Analysis of Variance
RER	Respiratory Exchange Ratio

## Appendix A

**Table A1:** Demographic and physical characteristics of all participants.

Participant No.	Sex	Age (Years)	Height (m)	Body Mass (kg)
1	M	25	1.78	72.6
2	M	26	1.72	68.3
3	M	25	1.83	78.6
4	M	27	1.86	80.4
5	M	25	1.85	65.1
6	M	31	1.75	78.2
7	M	26	1.69	65.5
8	M	28	1.83	84.4
9	F	32	1.68	62.1
10	F	34	1.67	71.4
11	F	27	1.72	63.4
12	F	31	1.57	56.5
13	F	28	1.69	60.6
Mean ± SD	8M/5F	28.08 ± 3.12	1.74 ± 0.09	69.78 ± 8.56

All participants were free of musculoskeletal/neurological disorders, could walk independently, and provided written informed consent. Exclusion criteria included recent lower-limb injury, exoskeleton contraindications, and inability to follow instructions.

**Table A2:** Hyperparameter settings of the five standalone neural network baseline models.

Model	Hyperparameters and Training Details
BPNN	Architecture: Input + FC + Output Hidden layers: 3 FC layers (64-128-64 units) Activation: ReLU Optimizer: Adam Learning rate: 5 × 10^−5^ Regularization: L2 penalty (λ = 1 × 10^−5^) Batch size: 16
FNN	Architecture: Input + FNN + Output Hidden layers: 2 FC layers (128-64 units) Activation: ReLU Optimizer: Adam Learning rate: 5 × 10^−5^ Regularization: L2 penalty (λ = 1 × 10^−5^) Batch size: 16
LSTM	Architecture: Input + LSTM + FC (linear) + Output LSTM hidden units: 128 FC layer units: 3 units Optimizer: Adam Learning rate: 5 × 10^−5^ Dropout: 0.4 Batch size: 16
RNN	Architecture: Input + RNN + FC + Output Hidden units: 128 FC layer units: 3 Optimizer: Adam Learning rate: 5 × 10^−5^ Dropout: 0.3 Batch size: 16
NTM	Architecture: Input + NTM + FC (linear) + Output Controller: LSTM (128 hidden units) Memory matrix: 128 × 40 (content-addressable) FC layer units: 3 units Read/Write heads: 1 head Optimizer: Adam Learning rate: 5 × 10^−5^ Batch size: 16

All models were evaluated using leave-one-subject-out (LOSO) cross-validation (13-fold). Training was performed with a maximum of 150 epochs and early stopping (patience = 10) based on validation loss. Within each training fold (n = 12 subjects), 20% of subjects (2–3 subjects) were held out as a validation set using subject-wise partitioning to ensure independence between training and validation data, preventing data leakage from the same participant. For the PDFM, the underlying NTM and LSTM sub-models retained their original hyperparameter configurations during fusion.
